# Rationale in the Use of Adjuvant Chemotherapy in pT3N0M0 Gastric Cancer Resected Patients. Comment on Chen et al. Prognostic Factors and the Role of Adjuvant Chemotherapy in Pathological Node-Negative T3 Gastric Cancer. *J. Pers. Med.* 2023, *13*, 553

**DOI:** 10.3390/jpm13060974

**Published:** 2023-06-09

**Authors:** Luigina Graziosi, Nicola Natalizi, Annibale Donini

**Affiliations:** Emergency and General Surgery, “Santa Maria della Misericordia” Hospital, University of Perugia, 06129 Perugia, Italy

I read with great interest the well-written and well-made study by Yi-Fu Chen et al. recently published in the “*Journal of Personalized Medicine*” [1].

Although surgery is the standard treatment for locally advanced gastric cancer (LAGC), the high rate of post-surgical recurrences is a critical issue for long-term patient survival [2].

For this reason, gastric cancer (GC) still remains one of the most common causes of death due to its malignancy and poor prognosis. 

In the past, most researchers asserted in their reviews that adjuvant treatment was not beneficial for prolonging survival after curative surgery [3].

Only since 2010, after large controlled studies, has the survival benefit of adjuvant chemotherapy (AC) been demonstrated, and has it become a standard of care after curative surgery and not an investigational approach.

To date it is unclear if there is a real advantage in N0 categories.

In this study, the authors did not clearly show the percentage of patients who underwent neoadjuvant chemotherapy (NAC). It is fundamental to know this because to date it is doubtful if AC is associated with improved overall survival (OS) in patients with LAGC who underwent NAC and subsequent gastrectomy. 

In fact, major studies reported that postoperative chemotherapy is omitted in nearly half of patients because of postoperative complications, poor nutritional status and functional decline, associating survival benefit only with pre-operative treatment. 

Lin J.X. et al. [4] demonstrated a significant interaction between Lymph Node Ratio (LNR) and AC in an NAC patient population: the receipt of AC has been associated with improved survival only in patients with an LNR of 9% or greater, probably because patients with a high LNR are at high risk of developing tumor recurrence after radical gastrectomy, and in need of additional AC to eliminate micrometastases.

Another issue to clarify in this paper is the type of lymphadenectomy performed in the resected population: a D2 dissection is essential for reaching an oncologically correct procedure, as the major guidelines recommended. 

AC could replace a suboptimal surgery when the surgical procedure is not associated with an adeguate lymphadenectomy, for example, when the lymph node retrieved is less than 16. Indeed, Jin et al. [5] demonstrated in their paper that patients with examined lymph nodes ≤ 15 could be particularly appropriate candidates for AC. 

Moreover, I would like to underline the conclusion of the authors emphasizing the existence of different molecular subtypes of gastric cancer that display unique biological behavior and different responsiveness to systemic treatment. 

TCGA classification evaluated and developed a prediction model for response to adjuvant chemotherapy showing that the EBV subtype appears to be associated with the best prognosis, MSI and CIN with a moderate prognosis, while GS showed the worst prognosis. When analyzing the molecular GC subtypes with respect to the benefit from AC, patients with CIN subtype showed the greatest benefit, while those with GS received no benefit, and only a moderate benefit was observed among patients with MSI subtype [6].

In addition, ongoing clinical trials are evaluating whether the administration of immune therapy in MSI-H GC in the adjuvant setting could cause a survival benefit.

I believe that the key is to identify clinical and molecular predictive and prognostic factors to select patients at higher risk of recurrence and patients that could receive a benefit from the adjuvant treatment. This would help clinicians to avoid chemotherapy for non-responder patients, avoiding useless toxicity.

## References

[1] Chen Y.-F., Chen M.-Y., Le P.-H., Chen T.-H., Kuo C.-J., Wang S.-Y., Huang S.-C., Chou W.-C., Yeh T.-S., Hsu J.-T. (2023). Prognostic Factors and the Role of Adjuvant Chemotherapy in Pathological Node-Negative T3 Gastric Cancer. J. Pers. Med..

[2] Torre L.A., Bray F., Siegel R.L., Ferlay J., Lortet-Tieulent J., Jemal A. (2015). Global cancer statistics, 2012. CA Cancer J. Clin..

[3] Hohenberger P., Gretschel S. (2003). Gastric cancer. Lancet.

[4] Lin J.X., Tang Y.H., Lin G.J., Ma Y.B., Desiderio J., Li P., Xie J.-W., Wang J.-B., Lu J., Chen Q.Y. (2022). Association of Adjuvant Chemotherapy With Overall Survival Among Patients With Locally Advanced Gastric Cancer After Neoadjuvant Chemotherapy. JAMA Netw. Open.

[5] Jin P., Ji X., Ma S., Kang W., Liu H., Li Y., Ma F., Hu H., Xiong J., Tian Y. (2021). Adjuvant Chemotherapy Indications for Stage I Gastric Cancer Patients with Negative Lymph Node. Clin. Res. Hepatol. Gastroenterol..

[6] Sohn B.H., Hwang J.-E., Jang H.-J., Lee H.-S., Oh S.C., Shim J.-J., Lee K.-W., Kim E.H., Yim S.Y., Lee S.H. (2017). Clinical Significance of Four Molecular Subtypes of Gastric Cancer Identified by The Cancer Genome Atlas Project. Clin. Cancer Res..

